# Pediatric Rash Illness Outbreak with Initial Positive Measles Immunoglobulin M Antibody Test Results — American Samoa, March–July 2023

**DOI:** 10.15585/mmwr.mm7345a3

**Published:** 2024-11-14

**Authors:** Ruth Stefanos, Sabrina Schatzman, Brian Wakeman, Kelley Raines, Lakshmi Radhakrishnan, Thomas D. Filardo, Stephen N. Crooke, Bettina Bankamp, R. Suzanne Beard, Terry Fei Fan Ng, Rachel L. Marine, Suxiang Tong, Adam Konrote, Astrid M. Johansson, Annette Fa’alevao Ilimaleota, Motusa Tuileama Nua, Sarah K. Kemble, Edward Desmond, Paul A. Rota, Janell A. Routh, W. Thane Hancock, David E. Sugerman, Magele Scott Anesi, American Samoa Response Group, American Samoa Response Group, Ronald Balajadia, Allison M. Brady, Christina J. Castro, Atefeh Paziraei Chamanzad, Tai-Ho Chen, Heather Colley, Janine Cory, Nathan E. Crawford, Brian D. Emery, Remedios B. Gose, Susette Japin, Peter Judicpa, Gimin Kim, Drew Kuwazaki, Elizabeth Lauvao, Yan Li, Josese Limaono, Sara Mercader, Nehalraza Momin, Romson Nuake, Angelynn Papu, Raijieli Rasekaseka, Maopa Raikabula, Adam C. Retchless, Shannon L. Rogers, Sun Bae Sowers, Ying Tao, Ashley Tippins, Alex Turner, Brandi Turner, Vasiti Uluiviti, Jing Zhang

**Affiliations:** ^1^Division of Viral Diseases, National Center for Immunization and Respiratory Diseases, CDC; ^2^Epidemic Intelligence Service, CDC; ^3^Laboratory Leadership Service, CDC; ^4^Division of Vector-Borne Diseases, National Center for Emerging and Zoonotic Infectious Diseases, CDC; ^5^Division of High-Consequence Pathogens, National Center for Emerging and Zoonotic Infectious Diseases, CDC; ^6^Division of Health Informatics and Surveillance, Center for Surveillance, Epidemiology, and Laboratory Services, CDC; ^7^American Samoa Department of Health; ^8^Hawaii Department of Health; ^9^Division of State and Local Readiness, Office for Readiness and Response, CDC.; Hawaii Department of Health; CDC; CDC; CDC; CDC; CDC; CDC; CDC; CDC; Hawaii Department of Health; American Samoa Department of Health; CDC; CDC; Hawaii Department of Health; American Samoa Department of Health; CDC; American Samoa Department of Health; CDC; CDC; American Samoa Department of Health; American Samoa Department of Health; American Samoa Department of Health; American Samoa Department of Health; CDC; CDC; CDC; CDC; CDC; CDC; CDC; Pacific Islands Health Officer’s Association, Honolulu, Hawaii; CDC.

**Summary**

**What is already known about this topic?**

In settings with low measles prevalence, measles immunoglobulin (Ig) M antibody testing results have low positive predictive value and can result in difficulties with interpreting results.

**What is added by this report?**

In 2023, the occurrence of positive measles IgM test results for two vaccinated children in American Samoa led to suspicion of a measles outbreak and resulted in declaration of a public health emergency and a mass vaccination campaign to improve coverage. Additional testing from these two children was not possible. Review of medical records and additional laboratory testing of subsequent persons under investigation confirmed alternative viral etiologies.

**What are the implications for public health practice?**

Confirmation of measles cases relies on a combination of clinical, serologic and molecular laboratory, and vaccination data. High measles vaccination coverage can prevent outbreaks.

## Abstract

On April 24, 2023, the American Samoa Department of Health (ASDoH) declared a public health emergency amid concern about a possible measles outbreak given low 2-dose vaccination coverage at the time. ASDoH had received two positive measles immunoglobulin (Ig) M test results after Flag Day festivities 1 week earlier from vaccinated children. ASDoH performed active case finding, took actions to mitigate transmission, and requested technical assistance from CDC. ASDoH implemented a vaccination campaign to improve suboptimal coverage. Confirmatory molecular testing of specimens from these initial persons under investigation (PUIs) was not possible, but subsequent testing of specimens from additional PUIs by Hawaii State Laboratories Division and CDC ruled out measles. In settings with low measles prevalence, measles antibody testing results have low positive predictive value and can lead to difficulties with interpreting results. Testing for additional pathogens revealed a variety of viruses known to cause common childhood viral exanthems. Both molecular and serologic testing should be performed for all suspected measles cases. To decrease the probability of false-positive IgM results, testing should be reserved for cases that meet the Council of State and Territorial Epidemiologists measles case definition, especially those in persons with no evidence of immunity and with a history of recent international travel. In addition, maintaining high measles vaccination coverage can prevent future outbreaks. 

## Investigation and Results

### Identification of Two Persons Who Received Positive Measles Immunoglobulin M Antibody Test Results

On March 23, 2023, an afebrile child aged 8 years who had received 2 doses of measles, mumps, and rubella (MMR) vaccine^†^ was evaluated in American Samoa, a remote unincorporated U.S. territory in the southern hemisphere (population approximately 50,000) (Figure) for a 3-day history of generalized pruritic rash. Nearly 20 days later, another afebrile child aged 4 years who also had received 2 doses of MMR vaccine was evaluated for a rash. Both children received a positive measles immunoglobulin (Ig) M test result from a commercial laboratory in California. The American Samoa Department of Health (ASDoH) received these results after Flag Day^§^ festivities, an island-wide event with large gatherings that occurred on April 17. Once a positive measles IgM test result was received, concern was raised that community measles transmission was already occurring.

**FIGURE:**
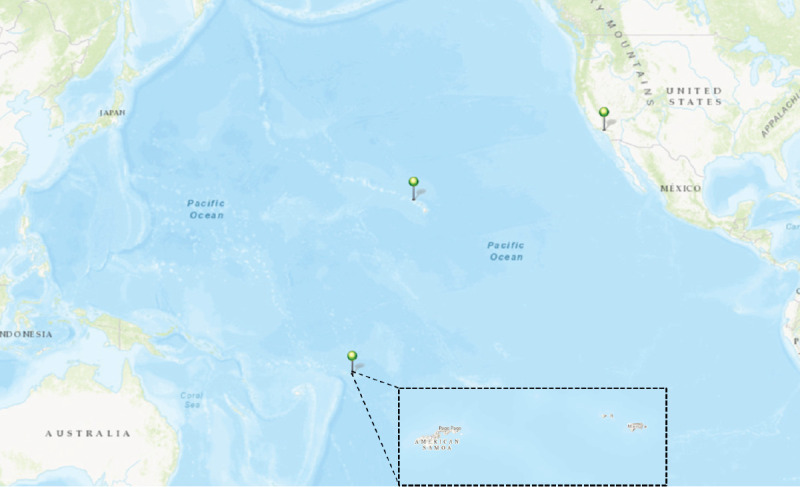
Geographic locations where non-CDC laboratory testing was performed for measles during a pediatric rash illness outbreak — American Samoa, California, and Hawaii, March–July 2023

In 2019, a large measles outbreak involving multiple Pacific Island countries occurred, including cases in American Samoa. High morbidity and mortality occurred in neighboring island country Samoa where vaccination coverage was low; nearly 6,000 measles cases and >80 measles-associated deaths occurred (*1*). Low 2-dose MMR vaccination coverage estimates (72%) among children aged 6 years in American Samoa in January 2023 (CDC, unpublished data, 2023) raised concern among health authorities about the potential for another large measles outbreak. Since 2019, the 21 Pacific Island Countries and Areas (PICs) of which American Samoa is a part, interrupted endemic measles transmission and were on track to achieve measles elimination (*2*).

### Declaration of Public Health Emergency

On April 24, 2023, 1 week after Flag Day, a public health emergency was declared in American Samoa. The ASDoH closed schools and child care centers, initiated a territory-wide MMR vaccination campaign, implemented travel restrictions, conducted active case finding, and requested technical assistance from CDC. This activity was reviewed by CDC, deemed not research, and was conducted consistent with applicable federal law and CDC policy.^¶^

### Identification of Persons Under Investigation

During March–July 2023, ASDoH evaluated 86 persons for measles (persons under investigation [PUIs]), including the two pediatric index patients. The median PUI age was 5.9 years, and 95% were aged <13 years (Table 1). Among all PUIs, illness among more than one half (51; 59%) did not meet the Council of State and Territorial Epidemiologists (CSTE) measles clinical case definition**; however, clinical suspicion of measles based on the characteristics and distribution of rash prompted laboratory testing for these children. Among the 35 children whose illness did meet the CSTE clinical case definition, 31 (86%) had received ≥1 MMR vaccine dose and 18 (all aged 1–12 years) had received 2 doses.

**TABLE 1:** Characteristics of persons under investigation for measles during a public health response, by age group — American Samoa, March–July 2023

Characteristic	No. (col. %)					
	Total* N = 86	Age group, yrs				
		**<1 n = 8**	**1–6 n = 43**	**7–12 n = 31**	**13–17 n = 3**	**≥18 n = 1**
Rash^†^	**71 (83)**	6 (75)	36 (84)	26 (84)	2 (67)	1 (100)
Rash plus fever^†^	**45 (52)**	4 (50)	26 (60)	14 (45)	1 (33)	0 (—)
MCC met, all^†^	**35 (41)**	3 (38)	24 (56)	8 (26)	0 (—)	0 (—)
MCC met, no MMR received^§^	**4 (5)**	3 (38)	1 (2)	0 (—)	0 (—)	0 (—)
MCC met, ≥1 MMR dose received^¶^	**31 (36)**	0 (—)	23 (53)	8 (26)	0 (—)	0 (—)
MCC met, ≥2 MMR doses received^¶^	**18 (21)**	0 (—)	10 (23)	8 (26)	0 (—)	0 (—)

**Abbreviations:** MCC = measles clinical criteria; MMR = measles, mumps, and rubella vaccine.

* During March 23–July 25, 2023, a total of 86 persons were investigated for measles. One child who was evaluated twice with different symptoms during this period was included based on symptoms reported at the first clinical encounter.

^†^ Council of State and Territorial Epidemiologists measles clinical criteria included rash and fever and at least one of the following: coryza, cough, or conjunctivitis (https://ndc.services.cdc.gov/case-definitions/measles-2013/). Three patients did not have electronic medical record notes, and information regarding cough was missing. Fever included subjective reports from parents and patients as well as measured temperatures during the health care encounter. Characteristics of rash and sequence of symptom occurrence was variable (i.e., some rashes were described as pruritic, or distribution of rash was not consistently described as generalized with spread from face down to the rest of the body).

^§^ No MMR vaccine dose was documented, or MMR vaccine was documented as having been given on or after April 25, 2023, after declaration of the public health emergency on April 24, 2023.

^¶^ MMR vaccine doses were included if administered on or before April 24, 2023. Doses administered to children aged <12 months were included.

### Laboratory Investigation

During the public health emergency, no validated molecular or serologic assays for measles were available in American Samoa, which resulted in the need for logistically challenging off-island testing. Off-island testing is affected by long travel times and limited flights from the island that delay receipt of specimens by laboratories. Nasopharyngeal (NP) swabs or throat swabs, the preferred specimen for detection of measles viral RNA, were not collected from the two initial PUIs, and therefore real-time reverse transcription-polymerase chain reaction (RT-PCR) testing was not performed. However, 62 specimens from PUIs identified later in the investigation were sent to the Hawaii State Laboratories Division for measles real-time RT-PCR testing. Among the 62 NP swabs and 52 serum samples sent to Hawaii, 37 (60%) NP swabs and 42 (81%) serum samples were forwarded to CDC for further testing, including 1) measles virus genotype A (MeVA) real-time RT-PCR to detect measles vaccine virus; 2) measles and rubella serology, including IgM and IgG; 3) IgG avidity testing for measles and rubella; and 4) measles plaque reduction neutralization (PRN) to determine baseline immunity and differentiate recent infection from vaccination. The CDC IgM capture enzyme immunoassay used for measles IgM testing has been found to be more sensitive and specific than indirect enzyme immunoassays (*3*). IgG avidity testing was performed to measure the overall strength of antigen-antibody binding to help distinguish recent from remote infection, and PRN is a functional assay that measures neutralizing antibodies. To identify additional pathogens, CDC performed enterovirus and parechovirus real-time RT-PCR and typing, pan-herpesvirus PCR, pan-erythrovirus PCR, agnostic metagenomic next-generation sequencing (NGS), and viral-enriched NGS on deidentified specimens.

All NP swabs tested at the Hawaii State Laboratories Division for measles using real-time RT-PCR were negative, with the exception of one from a recently vaccinated child (Table 2); based on MeVA testing, this result was consistent with measles vaccination 11 days before specimen collection. Measles IgM capture assay and IgG results were positive for four PUIs, which could indicate a recent or acute measles infection or recent measles vaccination. Two of the four PUIs with positive IgM capture and IgG test results also had high IgG avidity test results and low plaque reduction neutralization titers, suggesting false-positive IgM results (recent measles infection would elicit a high neutralizing antibody titer). One specimen from a PUI without reported MMR vaccination was also rubella IgM-positive and had low avidity for both measles and rubella, suggesting recent vaccination. ASDoH later confirmed an MMR dose 1 month before rash onset in their immunization registry. One IgM capture–positive specimen was not tested further. Thirty-two (87%) of 37 NP specimens tested at CDC were positive for any virus, including 19 (53%) for parvovirus B19. Other viruses (e.g., rhinovirus [12], human herpesvirus 6 [five], influenza B [five], respiratory syncytial virus [one], and SARS-CoV-2 [two]) were also identified. Among 14 (44%) of 32 specimens that yielded a positive virus test result, more than one pathogen was identified.

**TABLE 2:** Laboratory test results of persons under investigation for measles during public health response, by age group — American Samoa, March–July 2023

Laboratory test	No. of positive test results/Total no. (%), by age group, yrs					
	**<1**	**1–6**	**7–12**	**13–17**	**≥18**	All
Measles real-time RT-PCR*	0/7 (—)	1/28 (4)	0/25 (—)	0/1 (—)	0/1 (—)	**1/62 (2)**
Measles virus genotype A, RT-qPCR (MEVA)^†^	—^§^	1/1 (100)	—	—	—	**1/1 (100)**
CDC measles IgM^¶^	0/1 (—)	3/18 (17)	1/20 (5)	0/2 (—)	0/1 (—)	**4/42 (10)**
CDC measles IgG^¶^	0/1 (—)	16/18 (89)	20/20 (100)	2/2 (100)	1/1 (100)	**39/42 (93)**
CDC rubella IgM^¶^	0/1 (—)	3/18 (17)	2/20 (10)	0/2 (—)	0/1 (—)	**5/42 (12)**
CDC rubella IgG^¶^	0/1 (—)	17/18 (94)	19/20 (95)	2/2 (100)	1/1 (100)	**39/42 (93)**
Enterovirus and parechrovirus real-time RT-PCR**	4/6 (67)	3/15 (20)	3/15 (20)	—	0/1 (—)	**10/37 (27)**
**Deidentified specimen testing** ^††^						
Virus testing total^§§^ (any virus)	NA	NA	NA	NA	NA	**32/37 (87)**
Codetection	NA	NA	NA	NA	NA	**14/32 (44)**
Human parvovirus B19^¶¶^	NA	NA	NA	NA	NA	**19/36 (53)**
Rhinovirus	NA	NA	NA	NA	NA	**12/37 (32)**
Influenza^¶¶^	NA	NA	NA	NA	NA	**5/33 (15)**
HHV-6	NA	NA	NA	NA	NA	**5/33 (15)**
HHV-7	NA	NA	NA	NA	NA	**3/33 (9)**
SARS-CoV-2	NA	NA	NA	NA	NA	**2/33 (6)**
RSV	NA	NA	NA	NA	NA	**1/33 (3)**

**Abbreviations:** CLIA = Clinical Laboratory Improvement Amendments; IgG = immunoglobulin G; IgM = immunoglobulin M; HHV-6 = human herpesvirus 6, HHV-7= human herpesvirus 7; MeVA = measles virus genotype A; NA = not applicable; PCR = polymerase chain reaction; PUI = person under investigation; RSV = respiratory syncytial virus; RT-PCR = reverse transcription–polymerase chain reaction.

* One child aged 7–12 years was evaluated twice, and two specimens were collected. Specimens were not collected for all PUIs. As a result, test results are not available for one PUI aged <1 year, 15 aged 1–6 years, seven aged 7–12 years, and two aged 13–17 years.

^†^ Measles vaccine strain was detected in the only positive measles real-time RT-PCR specimen from a PUI vaccinated against measles 11 days before specimen collection.

^§^ Dashes indicate that there were no specimens tested in that age range.

^¶^ Specimens that were positive for both IgM and IgG for measles or rubella were further evaluated with measles and rubella IgG avidity and measles plaque reduction neutralization testing.

** Positive enterovirus and parechrovirus RT-PCR specimens were further evaluated, and all were determined to be rhinovirus types.

^††^ Specimens were deidentified before non-CLIA testing, and results cannot be age stratified.

^§§^ Summary of viruses that were identified from enterovirus and parechrovirus typing, pan-herpesvirus PCR, pan-erythrovirus PCR, and next generation sequencing including pan-viral enrichment and metagenomics sequencing methods. Multiple viruses were detected in 14 specimens.

^¶¶^ One specimen was depleted after enterovirus and parechrovirus typing, and three specimens were depleted after pan-erythrovirus PCR testing.

## Public Health Response

The public health emergency ended on June 8, 2023. To improve communication between response team members in American Samoa, including laboratorians, clinicians, and epidemiologists, a table-top exercise was facilitated. Continuing medical education lectures were offered to clinicians to review measles diagnostic criteria and appropriate testing. Initial efforts to create a measles response manual and an improved case-based surveillance system are ongoing. Establishment of a syndromic surveillance system at ASDoH, including data from the Lyndon B. Johnson Tropical Medical Center, the only hospital on the island, and other health care facilities in the region, were already in development before this investigation and continued opportunities were explored through the National Syndromic Surveillance Program. Efforts to support laboratory capacity building were also explored and ongoing at ASDoH. These efforts include providing a measles serological IgM capture enzyme-linked immunosorbent assay to increase assay specificity, laboratory training and test validation panels, and continued assistance to bring molecular testing online.

## Discussion

The receipt of two positive measles IgM test results by fully vaccinated children with rash illnesses whose clinical signs and symptoms did not meet the CSTE clinical case definition for measles resulted in declaration of a public health emergency in American Samoa. A widespread measles outbreak with high morbidity and mortality on a neighboring island 4 years earlier, coupled with suboptimal measles vaccination coverage and recent large public gatherings, raised concern about the possibility of a large outbreak and prompted these public health actions.

In a person who has not received measles vaccine, signs and symptoms of disease are typically 3–5 days of fever along with cough, coryza, or conjunctivitis, followed by a generalized, descending, maculopapular rash. The differential diagnosis includes Fifth Disease (caused by parvovirus B19), hand-foot-and-mouth disease (most commonly caused by coxsackievirus, an enterovirus), roseola or exanthem subitum (caused by human herpesvirus 6), human herpesvirus 7, rubella and other viral exanthems.

CDC recommends that health care providers collect NP or throat swabs for molecular and peripheral blood for serologic testing for all suspected cases of measles (i.e., cases of febrile rash illness).^††^ Serologic assays are important for ascertaining immunity to measles as well identification of actual cases, including those that occur in vaccinated persons (*4**,**5*). However, as countries achieve or approach elimination status, the positive predictive value of IgM testing for measles (i.e., the likelihood that a positive test indicates the presence of disease) decreases in low incidence settings and increases the risk for false-positive results (*6*). In addition, sensitivity and specificity of measles IgM assays can vary because of cross-reactivity to pathogens associated with common childhood viral exanthems, with parvovirus B19 particularly implicated in some assays,^§§^ thereby creating the potential for false-positive results in areas with high measles vaccination coverage and low disease incidence (*7*).

Some persons experience signs and symptoms associated with measles, including rash, after measles vaccination, and RT-PCR as well as IgM and IgG and serologic testing cannot distinguish vaccine-associated rash from measles illness; measles vaccine strain viral RNA has been detected months after vaccination (*8*). CDC’s MeVA testing can distinguish vaccine-associated rash illness from infection with wild measles virus because it detects only measles vaccines strains. In a suspected outbreak setting, MeVA testing can distinguish vaccine reactions from actual measles cases even as vaccination campaigns are ongoing (*9*).

In some cases, additional serologic assays can also be used to further classify cases. For example, IgG avidity assays can be performed on IgG-positive specimens to aid in distinguishing recent immunity (resulting in low avidity) from immunity that developed in the past (resulting in high avidity). PRN is considered the benchmark serologic assay to determine the presence of measles neutralizing antibodies (*10*).

Specimens from the initial PUIs in this investigation were unavailable for further laboratory testing. Clinical and laboratory findings, including reference test findings from 84 other PUIs, suggest that rash illnesses might have been caused by other viral pathogens, including parvovirus B-19, which can cross-react with measles IgM testing. As countries approach measles elimination, enhancing local capacity for molecular and serologic diagnostic testing is critical to rapidly discriminating between actual measles cases, vaccine-associated rash illness, and cases in previously vaccinated persons so that recommended public health measures can be implemented.

After this investigation, improvements at ASDoH in partnership with the Lyndon B. Johnson Tropical Medical Center in case-based and syndromic surveillance and laboratory serologic and molecular capacity are in progress in American Samoa. These efforts will support future public health outbreak response efforts for measles and might serve as a model for other Pacific Islands. Physicians should be cautious about ordering and interpreting measles IgM testing when a patient’s clinical features do not meet the CSTE measles case definition, especially in settings where measles prevalence is low, patients have documentation of measles immunity, and there is no history of recent international travel. In settings of low vaccination coverage, physicians and public health authorities should take appropriate infection control and mitigation measures while awaiting confirmatory test results. Achieving and maintaining high coverage with measles-containing vaccine through routine vaccination programs is critical to preventing measles outbreaks and achieving measles elimination.

## Footnotes

† Per the U.S. routine MMR vaccination schedule, children are usually considered fully vaccinated against measles after receipt of 2 MMR vaccine doses at ages 12–15 months and 4–6 years. https://www.cdc.gov/vaccines/vpd/mmr/public/index.html

§ The American flag was officially raised in American Samoa for the first time on April 17, 1900, and Flag Day is celebrated each year on April 17 in commemoration of this historic event.

¶ 45 C.F.R. part 46.102(l)(2), 21 C.F.R. part 56; 42 U.S.C. Sect. 241(d); 5 U.S.C. Sect. 552a; 44 U.S.C. Sect. 3501 et seq.

** A generalized maculopapular rash with fever and at least one of the following: cough, coryza, or conjunctivitis. https://ndc.services.cdc.gov/case-definitions/measles-2013/

††
https://www.cdc.gov/measles/hcp/clinical-overview/?CDC_AAref_Val=https://www.cdc.gov/measles/hcp/index.html

§§ In addition, cross reactivity with another similar virus or rheumatoid factor can cause false-positive IgM test results.

## References

[1] Craig AT, Heywood AE, Worth H. Measles epidemic in Samoa and other Pacific islands. Lancet Infect Dis 2020;20:273–5. 10.1016/S1473-3099(20)30053-0 32112752

[2] World Health Organization, Regional Office for the Western Pacific. Tenth Annual Meeting of the Regional Verification Commission for Measles and Rubella Elimination in the Western Pacific, Manila, Philippines, September 12–16, 2022: meeting report. Manila, Philippines: World Health Organization, Regional Office for the Western Pacific; 2022. https://iris.who.int/handle/10665/365590

[3] Sowers SB, Anthony K, Mercader S, Performance characteristics of six immunoglobulin M enzyme-linked immunosorbent assays used for laboratory confirmation of measles. J Clin Microbiol 2022;60:e0122722. 10.1128/jcm.01227-22 36409098 PMC9769589

[4] Mercader S, Dominguez A, Torner N, Classification of measles breakthrough cases in an elimination setting using a comprehensive algorithm of laboratory results: why sensitive and specific IgM assays are important. Int J Infect Dis 2021;112:21–4. 10.1016/j.ijid.2021.09.004 34508861 PMC11329164

[5] Sowers SB, Rota JS, Hickman CJ, High concentrations of measles neutralizing antibodies and high-avidity measles IgG accurately identify measles reinfection cases. Clin Vaccine Immunol 2016;23:707–16. 10.1128/CVI.00268-16 27335386 PMC4979181

[6] Bolotin S, Lim G, Dang V, The utility of measles and rubella IgM serology in an elimination setting, Ontario, Canada, 2009–2014. PLoS One 2017;12:e0181172. 10.1371/journal.pone.0181172 28850604 PMC5574571

[7] Hiebert J, Zubach V, Charlton CL, Evaluation of diagnostic accuracy of eight commercial assays for the detection of measles virus-specific IgM antibodies. J Clin Microbiol 2021;59:e03161–20. 10.1128/JCM.03161-20 33731415 PMC8315954

[8] McMahon J, Mackay IM, Lambert SB. Measles vaccine virus RNA in children more than 100 days after vaccination. Viruses 2019;11:636. 10.3390/v11070636 31295941 PMC6669751

[9] Martin KG, Banerjee E, McMahon M, Identifying vaccine-associated rash illness amidst a large measles outbreak: Minnesota, 2017. Clin Infect Dis 2020;71:e517–9. 10.1093/cid/ciaa168 32067029

[10] Cohen BJ, Parry RP, Doblas D, . Measles immunity testing: comparison of two measles IgG ELISAs with plaque reduction neutralisation assay. J Virol Methods 2006;131(2):209–12. 10.1016/j.jviromet.2005.08.001 16188328

